# Health financing policies in Sub-Saharan Africa: government ownership or donors’ influence? A scoping review of policymaking processes

**DOI:** 10.1186/s41256-017-0043-x

**Published:** 2017-08-08

**Authors:** Lara Gautier, Valéry Ridde

**Affiliations:** 1 Department of social and preventive medicine, School of Public Health, Université de Montréal, Montréal, Québec Canada; 2 Public Health Research Institute (IRSPUM), Université de Montréal, Montréal, Québec Canada; 3 Centre d’Etudes en Sciences Sociales sur les Mondes Africains, Américains et Asiatiques, Université Paris Diderot-Paris VII, Sorbonne Paris Cité, Paris, France

## Abstract

**Background:**

The rise on the international scene of advocacy for universal health coverage (UHC) was accompanied by the promotion of a variety of health financing policies. Major donors presented health insurance, user fee exemption, and results-based financing policies as relevant instruments for achieving UHC in Sub-Saharan Africa. The “donor-driven” push for policies aiming at UHC raises concerns about governments’ effective buy-in of such policies. Because the latter has implications on the success of such policies, we searched for evidence of government ownership of the policymaking process.

**Methods:**

We conducted a scoping review of the English and French literature from January 2001 to December 2015 on government ownership of decision-making on policies aiming at UHC in Sub-Saharan Africa. Thirty-five (35) results were retrieved. We extracted, synthesized and analyzed data in order to provide insights on ownership at five stages of the policymaking process: emergence, formulation, funding, implementation, and evaluation.

**Results:**

The majority of articles (24/35) showed mixed results (i.e. ownership was identified at one or more levels of policymaking process but not all) in terms of government ownership. Authors of only five papers provided evidence of ownership at all reviewed policymaking stages. When results demonstrated some lack of government ownership at any of the five stages, we noticed that donors did not necessarily play a role: other actors’ involvement was contributing to undermining government-owned decision-making, such as the private sector. We also found evidence that both government ownership and donors’ influence can successfully coexist.

**Discussion:**

Future research should look beyond indicators of government ownership, by analyzing historical factors behind the imbalance of power between the different actors during policy negotiations. There is a need to investigate how some national actors become policy champions and thereby influence policy formulation. In order to effectively achieve government ownership of financing policies aiming at UHC, we recommend strengthening the State’s coordination and domestic funding mobilization roles, together with securing a higher involvement of governmental (both political and technical) actors by donors.

**Electronic supplementary material:**

The online version of this article (doi:10.1186/s41256-017-0043-x) contains supplementary material, which is available to authorized users.

## Background

Over the past few years, and especially since the publication of the 2010 World Health Report (WHR) [1], universal health coverage (UHC) has generated a wide consensus at the international level [2–5]. As per World Health Organization (WHO)'s online factsheet, UHC aims at reaching a balance between extending access to healthcare services to all people, ensuring affordability for all people, and improving quality of care [6]. In the 2010s, there was a more explicit push for UHC, to the point of being officially included as an official target of the Sustainable Development Goals for 2016–2030 [7]. Initially focusing on financing mechanisms, this global movement [8, 9] produced a restricted understanding of UHC and resulted in overlooking issues related to equity and quality of care [5]. The rise on the international scene of advocacy for UHC was thus accompanied by the promotion of new health financing policies (e.g., health insurance, user fee exemption, and results-based financing), which donors presented as relevant instruments for achieving UHC [10].

### Financing policies aiming at UHC

Health insurance in its various forms (community-based health insurance, national insurance schemes, etc.) was introduced in the African landscape about 20 years ago [11]. Health insurance aims to improve financial access to health care (of those affiliated) through a reduction of patients’ direct payments. Another strategy to increase demand is the abolition of the direct payment for care (or “user fee exemption”) for certain services or specific categories of population. By curbing out-of-pocket expenditures, this strategy is set to improve financial access to health services. It emerged in the mid-2000s in response to the negative effects of cost recovery established in the early 1990s under the leadership of the World Bank and UNICEF [12]. Results-based financing (RBF) “encompasses the entire range of financial incentive approaches on both the demand and the supply sides” [13], including, mainly: conditional cash transfers (CCTs), performance-based financing (PBF), and performance-based contracting (i.e., a form of supply-side incentive used by donors). CCTs are demand-side incentives, providing cash rewards to target populations for “consuming certain social services” [13]. Globally, PBF emerged in the late 2000s around the idea – promoted by donors [14] – that it would help to improve access to quality health services. PBF is based on the transfer of financial resources conditional to achieving pre-agreed targets relating to health providers’ or managers’ performance [13]. International organizations have framed PBF in the language of “strategic purchasing” for UHC [15, 16]. The promotion of UHC has led to the increased implementation of these three financing policies since 2010, particularly in Sub-Saharan Africa [17] – this is the reason for choosing said region as the geographical focus of this review.

These policies have generated mixed results in terms of increased use and quality of healthcare [18–21]. Ownership, defined as “an attitude of accepting responsibility for something and taking control of how it develops” [22], can yield positive results. For instance, it can aid in eliminating barriers to access to care [23]. The mixed results achieved by these policies may be attributable to their “top-down” nature, reflecting the possibility that donor-driven policies do not necessarily fit local contexts [24], and therefore lack buy-in from governments.

### Genesis of the concept of “ownership”

Global health decision-making primarily involves a wide variety of donors [25] including bilateral, multilateral agencies, and international financial institutions (IFIs), as well as non-state actors (inter alia, non-governmental organizations and private-for-profit entities). In this context, the political voice and power of developing nations’ governments tend to be limited [26–28]. The high dependence of Sub-Saharan African countries to foreign aid reinforces this tendency. In 2008 external sources provided more than 20% of total health expenditure in nearly half (48%) of the 46 countries in the WHO African Region [29]. Consequently, various development actors and scholars began to call for the reshaping of global governance towards a better inclusion of developing countries, or “Southern” actors [25, 30, 31]. The idea materialized around the promotion of “ownership” by countries from the South [32, 33].

Originally, for IFIs, “ownership” implied limiting the resistance opposed by Southern governments as well as enhancing their liability so as to ensure good behavior of debitors [34]. Ownership of the policy by debitor governments became indispensable to the IFIs who needed a return on investments [35]. The prospect clearly remained that of the donors, whose priority was to “hedge their own political risk” rather than to foster inclusion of recipient countries in policymaking [36].

In spite of their criticism of conditionality, scholars perceived the IFI’s approach as a first step towards actual ownership by Southern governments [32, 37]. They refer here to “national ownership” and “government ownership”. Woll argues that national ownership “implies a broad-based consensus in a [country’s] society at large”, while “government ownership” means the government effectively controls the content, implements programs, and secures the commitment of political and administrative elites [38]. The concept of government ownership applies to Foucault and the construction of biopower in the South [28]. Yet this concept did not win donors’ support [36]. Indeed, the 2005 Paris Declaration for Aid Effectiveness endorsed the concept of “country ​​ownership”, whereby recipient governments were simply invited to “exercise leadership in developing and implementing their national development strategies”, establish their own systems for donor coordination, and accept only assistance that meets their needs [39]. Exercising leadership is only a mild version of taking actual control over a policy. Importantly, “country ownership” introduces a new ingredient: consultation with civil society and non-governmental organizations (NGOs) [40]. However, in the absence of any guidance “as to who specifically should be involved in what activities” [41], such participatory process led to dissolving responsibilities and diminishing the role of governments. Today, country ownership is still depicted as an inconsistent and “underspecified” term [41]. Some scholars even consider it “misleading” [42]. Because the definition of “government ownership” is more straightforward and in accordance with our vision of more equitable global public health governance, we chose to use this concept in our review.

### Definition of the research question

Improving government ownership is one key strategy envisioned by academics to rebalance global governance for health [25, 32]. Indeed, government’s buy-in has obvious implications for the success of global health policies [43, 44]. In this research, the terms "government" and "State" are used interchangeably: they refer to the highest level of political power in a given country. There is little knowledge about what the concept of ownership means for the recipient countries themselves, and how they put it into practice [36]. Based on a rapid review of the concept, we identify four main indicators of government ownership: political commitment by demonstrating leadership at the highest levels of government [34, 45], effective engagement of technical levels of government [46, 47], ability of the government to coordinate international actors within public bodies [45, 48–50], and the government’s mobilization of domestic resources to finance the policy in the long term [48, 51]. Because the UHC goal will continue to gain global traction, we critically assessed the extent to which recipient countries have owned financing policies aiming to achieve this goal. We examined the presence of indicators of government ownership at different phases of decision-making for policies aiming at UHC (detailed below) and investigated whether and how donors influenced this process [52, 53].

## Methods

Mays and colleagues assert that scoping reviews are useful to “map key concepts underpinning a research area […], and [are rapidly] undertaken as stand-alone projects in their own right, especially where an area is complex or has not been reviewed comprehensively before” [54]. This type of literature reviews adequately fits our research interests: we investigated how the concept of ownership materializes along the policymaking process. We performed a scoping review which takes the form of a transparent mixed studies review of the empirical (quantitative, qualitative, and mixed methods designs) peer-reviewed literature in English and French, from January 2001 (following the implementation of the Millennium Development Goals that entailed many health reforms including those presented above in 1.1) to December 2015 (15-year timespan), on Sub-Saharan African (SSA) governments’ ownership of health financing policies to attain UHC. Four major scientific databases were looked upon: Medline/Pubmed, EBSCOHost, and Web of Science (for English literature), and CAIRN database (for French literature).

We followed the step-by-step approach for performing scoping reviews developed by Arksey & O’Malley in 2005 [55] and improved in 2010 by Levac, Colquhoun, and O’Brien [56].

### Search strategy

We provided the full list of keywords as Additional file 1. We sought to use as many variances as possible of the keywords (e.g., synonyms of “country ownership”) in order to make sure that we would cover the relevant papers.

### Selecting the relevant papers

The first author screened initial results’ titles and abstracts and excluded those that did not fall in our inclusion criteria (Table 1).

**Table 1:** List of inclusion criteria

Inclusion criteria
Peer-reviewed papers that were published between January 2001 and December 2015
Peer-reviewed papers examining policymaking processes implemented after 2000
Full text of peer-reviewed papers available in French- or English-language
Peer-reviewed papers that specifically targeted one or several SSA countries
Peer-reviewed papers focusing on public policies of health insurance (community or national schemes), user fee exemption, and results-based financing
Peer-reviewed papers showing a strong (i.e., main topic of the paper) or moderate (i.e., secondary topic or sub-section of the paper) focus on strategies for government ownership of policies aiming at UHC
Peer-reviewed papers with a strong methodological background and/or providing useful findings directly related to government ownership

### Charting the data

Once we selected the final results, we developed a data extraction form on Excel as a systematic tool to collect the relevant data for our study (see Table 2). The form is available upon request.

**Table 2:** Categories of information in the data extraction form

Author and year of publication
Type of paper (original research article, systematic review, conference proceedings), focus country(ies)
Magnitude of the focus on country ownership (strong: main topic of the paper, or moderate: secondary topic or sub-section of the paper)
Focus health financing polic(ies) (user fee exemption, health insurance,…)
Description of the main topic of investigation
Study design (qualitative, quantitative, mixed methods)
Methods used (case study, stakeholders analysis, literature review, etc.)
Findings at the emergence stage
Findings at the formulation stage
Findings at the funding stage
Findings at the implementation stage
Findings at the evaluation stage
Results on overall government ownership
Discussion and observations notes

### Collating, summarizing and reporting the results

Based on Pluye & Hong’s methodology for conducting mixed studies review and classification of analytical designs [57], we chose the convergent design. All included studies were synthesized qualitatively by the first author. The framework developed by Rocher [58] guided our analysis of the results. Rocher’s original framework, largely inspired by the traditional public policy cycle [59], covers actors involved in the following steps of policymaking: conceptualization, promotion, formulation, funding, and implementation. For the sake of convenience, we merged conceptualization and promotion, and added another stage: policy evaluation – which is traditionally part of the policy cycle, yet rarely investigated in global health policy [60]. Our final themes are the following: emergence (1), formulation (2), funding (3), implementation (4), evaluation (5). The five themes are defined in Table 3. Despite the limitations of the heuristic framework [61], which conceptualized policymaking as a linear process, we chose to use this approach because it enabled us to easily identify indicators of government ownership and/or donors’ influence at each stage.

**Table 3:** Definitions of the five policymaking stages

Policymaking stage	Definition	Matching indicator of government ownership
Emergence	The moment where a predictable or unpredictable “policy window” is seized by decision makers for initiating a strategy aiming at addressing an emerging and sometimes burning issue [62]. Some authors refer to it as the stage of “agenda-setting”.	*- Political will and leadership demonstrated at the highest level of government*
Formulation	The stage where the content of the policy is defined	*- Political will and leadership demonstrated at the highest level of government* *- Effective engagement at technical and operational levels of government* *- Capacity to act and coordinate actors within public agencies*
Funding	The step where financial provisions are made available to the implementing structure in charge of the policy implementation.	*- Effective engagement at technical and operational levels of government* *- Mobilization of national resources for contributing to finance the policy*
Implementation	Ability of the State to lead and coordinate the operationalization of the policy.	*- Effective engagement at technical and operational levels of government* *- Capacity to act and coordinate actors within public agencies* *- Mobilization of national resources for contributing to finance the policy*
Evaluation	Appreciating policy processes and outcomes [63].	*- Effective engagement at technical and operational levels of government* *- Capacity to act and coordinate actors within public agencies*

We searched for occurrence of one or more core indicators of government ownership at each policymaking stage. Based on this investigation, we assessed each phase as owned (evidence of ownership indicators based on selected papers’ findings), not owned (lack of evidence), or owned to a certain extent (mixed evidence). Mixed evidence meant that there were some indicators of ownership at a given policy stage but not all of them.

Finally, we looked at the overall ownership of policies’ decision-making process by combining the results of each reviewed stage. The results were assessed based on the five policymaking themes and this last “overall ownership” theme (i.e., six themes in total), which are also represented in the categories of our data extraction as shown above.

## Results

Out of the 848 papers (of which, 257 duplicates were removed) found on scientific databases, we pre-selected 76 papers based on their title and abstract. After reading their full text, we excluded 41 papers because their content did not match our review goals. Details of exclusion are provided in Table 4.

**Table 4:** List of exclusion criteria applied to select relevant papers

Exclusion criteria	
Content outside of scope (*N* = 18)	Exclusive focus on policy outcomes [64–75] Exclusive focus on vertical programs dealing with HIV/AIDS, malaria and tuberculosis [76–81]
Review of all health financing policy options without specific content on ownership (*N* = 6)	[82–87]
Analysis of public’s perceptions about health financing policies (*N* = 5)	[88–92]
Review of options for fiscally sustainable policies (*N* = 3)	[93–95]
Lack of substantial content on ownership (*N* = 6)	[96–101]
Full manuscript inaccessible (*N* = 1)	[102]
Short comments, not empirical papers (*N* = 2)	[103, 104]

**Fig. 1:**
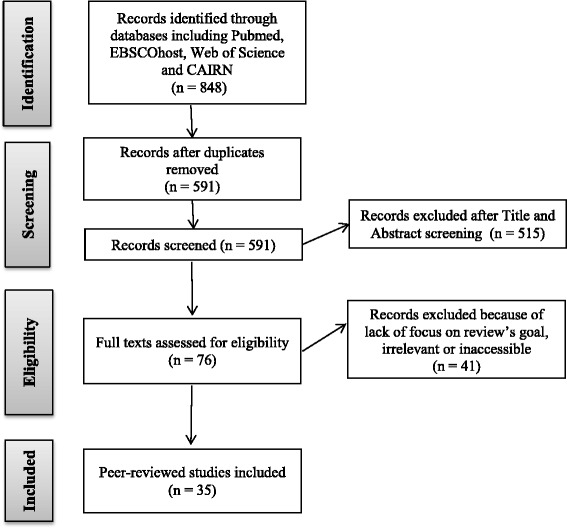
“PRISMA” flow diagram. Legend: Adapted from [158]

We selected a total of 35 (30 English-language and five French-language) peer-reviewed papers (see Fig. 1).

### Information on background and methods of the selected papers

Details about country and policy representation in the literature are available in Table 5. In country-specific papers, the most frequent countries of study were Ghana [105–110] and Burkina Faso [107, 110–114] (each *N* = 6).

**Table 5:** Distribution of financing policies and countries addressed in each paper

Article details	User fee exemption or reduction	National health insurance	PBF	PBF & user fee exemption or reduction	National subsidy for obstetric care	Community insurance	All UHC financing policies
Agyepong et al. 2008		Ghana					
Atim 2011							Africa (all countries)
Basaza et al. 2013		Uganda					
Chimhutu et al. 2014			Tanzania				
Chirwa et al. 2013	Malawi						
Falisse et al. 2012			Burundi				
Falisse et al. 2014				Burundi			
Fox et al. 2014				DRC			
Gilson et al. 2003		South Africa Zambia					
Gilson et al. 2012		South Africa Tanzania					
Kajula et al. 2004	Uganda						
Kirigia & Diarra-Nama 2008							Countries of the WHO African region
Kusi-Ampofo et al. 2015		Ghana					
Manitu et al. 2015			DRC				
Masiye et al. 2010	Zambia						
Mbaye et al. 2013	Senegal						
McIntyre et al. 2013	Malawi	Nigeria Tanzania					
Meda et al. 2011					Burkina Faso		
Meessen 2011	Burkina Faso BurundiGhana Liberia Senegal Uganda						
Nabyonga-Orem et al. 2014	Uganda						
Nyandekwe et al. 2014						Rwanda	
Olivier de Sardan et al. 2012	Burkina Faso Mali Niger						
Onoka et al. 2014		Nigeria					
Paul et al. 2014			Benin				
Peerenboom et al. 2014				Burundi			
Ponsar et al. 2011	Mali						
Ridde 2011					Burkina Faso		
Ridde et al. 2012	Benin Burkina Faso Mali Niger Togo Senegal						
Rusa et al. 2009			Rwanda				
Seddoh & Akor 2012		Ghana					
Thomas & Gilson 2004	South Africa						
Torbica et al. 2014	10 countries of West Africa						
Witter et al. 2013a	Sudan						
Witter et al. 2013b	Ghana						
Ye et al. 2014			Burkina Faso Ghana Tanzania				
Total	14	8	6	3	2	1	2

Performance-based financing (PBF) is the only RBF policy represented in our selection. The majority of selected articles had a strong emphasis on government ownership of policies aiming at UHC (*N* = 22). The remaining 13 articles had only a moderate focus on this theme but still provided useful insights for this review. Table 6 provides additional background information.

**Table 6:** Information about publication period, types of papers, and study design

Publication period	
Papers published between 2011 and 2015	*N* = 28
Papers published between 2006 and 2010	*N* = 4
Papers published between 2001 and 2005	*N* = 3
Types of papers	
Original research articles	*N* = 32
Reviews	*N* = 2
Conference report	*N* = 1
Study design	
Qualitative methods	*N* = 22
Quantitative methods	*N* = 10
Mixed methods	*N* = 3

Data collection included semi-structured in-depth interviews, focus group discussions, participant observation, and documentation review. When provided by the authors, analytical design was mostly stakeholders’ analysis (*N* = 4) [115–118], specific theory-based frameworks [106, 108, 119], or self-constructed frameworks [120]. Two quantitative papers were case-control studies using descriptive statistics with data obtained from questionnaire-based surveys [121, 122]. A third paper reviewed quantitative outcome records without using statistical analysis [123]. Despite their low methodological quality (i.e., very few details given about data collection and analysis), we included a couple of papers because they still provided useful information for our review [106, 111].

As shown in Table 7, only four papers looked into each of the five policymaking phases. Most papers addressed emergence, formulation, and funding phases.

**Table 7:** Policymaking stages addressed in selected papers

Article details	Emergence	Formulation	Funding	Implementation	Evaluation
Agyepong et al. 2008		X	X	X	
Atim 2011		X	X		
Basaza et al. 2013	X	X	X	X	
Chimhutu et al. 2014	X		X		
Chirwa et al. 2013	X	X	X	X	X
Falisse et al. 2012	X	X	X	X	X
Falisse et al. 2014	X	X			
Fox et al. 2014	X	X	X	X	
Gilson et al. 2003	X	X	X	X	
Gilson et al. 2012	X	X	X		
Kajula et al. 2004	X	X	X	X	
Kirigia & Diarra-Nama 2008			X		
Kusi-Ampofo et al. 2015	X				
Manitu et al. 2015	X		X	X	X
Masiye et al. 2010	X	X	X	X	
Mbaye et al. 2013	X	X	X		
McIntyre et al. 2013	X	X	X	X	
Meda et al. 2011			X	X	X
Meessen 2011	X	X	X	X	X
Nabyonga-Orem et al. 2014	X		X		
Nyandekwe et al. 2014	X	X	X	X	
Olivier de Sardan et al. 2012	X	X	X	X	
Onoka et al. 2014	X	X		X	
Paul et al. 2014	X	X	X	X	
Peerenboom et al. 2014		X	X	X	X
Ponsar et al. 2011		X	X	X	
Ridde 2011	X	X	X	X	X
Ridde et al. 2012	X	X	X		
Rusa et al. 2009	X	X		X	X
Seddoh et Akor 2012	X	X			
Thomas & Gilson 2004	X	X			
Torbica et al. 2014	X	X	X	X	
Witter et al. 2013a	X	X	X		
Witter et al. 2013b	X	X	X	X	
Ye et al. 2014	X		X		
Total	29	28	29	22	8

### Government ownership at the emergence stage

In general, ownership at the policy’s emergence stage turned out to be very diverse, with evidence of leadership expressed at the highest level in some countries in the case of health insurance, recurring illustrations of political instrumentalization in view of upcoming elections in the case of user fee exemption, and some evidence of influence by donors in the case of PBF. In some instances, like in Tanzania and Burundi, authors reported that policy emergence came from both the government and the donors [116, 120]. However, because we cannot identify how the common decision was reached, we can hardly talk about full ownership of the emergence phase.

Our results showed that governments were instigating the emergence of health insurance and user fee exemption, and that other players (including donors) often played a secondary role in SSA countries. In the case of user fee exemption, some authors noted that high-level politicians made the decision not for demonstrating leadership of the State in protecting its people but for “electoral benefits attached” to free health care [107]. Offering a package of free healthcare services represented a highly visible measure serving political campaigns. In addition, the decision to remove user fees was often controversial: in Burundi, Liberia and Uganda, the decision was made hastily and without any prior input from technical experts at the Ministry of Health [107]. Similar patterns characterized the emergence stage in Mali and Niger [112].

Emergence of health insurance followed a more gradual path, with a history of reforms on insurance in Ghana and Rwanda [105, 125]. In the case of Nigeria, high-level leadership came when a new minister of health “effectively managed stakeholders’ interests and galvanized their support to advance the policy” [118]. In South Africa, the changing balance of power within the major political party, the African National Congress, represented a window of opportunity for undertaking the reform [116].

In one paper “political commitment” was perceived as the most important criterion influencing policymaking on user fee abolition or reduction; whereas “international pressure” was one of the least important criteria [126]. Nevertheless, in three papers there were indications that external players played a role in pushing for user fee exemption [107, 108, 112]. Donors in many West African countries influenced the policy idea, even if it happened within the government. In both Ghana and Senegal, there were reports of “donor pressure” for policymaking [107, 108]. In Burkina Faso and Niger, the World Bank put pressure on the governments for introducing user fee exemption [112]. In Mali, the need to be eligible to grants provided by the Global Fund prompted the decision [112]. Interestingly, authors of another paper reported the generation and use by donors of a variety of evidence to “push” the user fee exemption policy in Uganda. This report suggests instrumentalization of knowledge-based resources for convincing governments [127].

As for performance-based financing (PBF), at the beginning neither Rwanda [123] nor Burundi [121, 124] were ﻿described as ﻿showing government﻿al ownership: international NGOs – latter with support from multilateral organizations (e.g., the World Bank) – implemented the policy through pilot projects. In Burundi, inspiration came from “the 1993 World Bank report ‘Investing in Health’, the agenda of the MDGs […], and the PBF experience in neighboring Rwanda that was then described as ‘encouraging’” [121]. Ye and colleagues also cited the potential of PBF to accelerate the achievement of MDGs as main driver for policy introduction [110]. In Rwanda, authors mentioned the 56th World Health Assembly [123]. In Benin, donors played an instrumental role in convincing the government of the value of PBF [128], and therefore in setting the policy window in favor of piloting and then scaling-up PBF. In DRC, there was no information on the role(s) played by the State [129].

### Government ownership at the policy formulation stage

Overall, government ownership at the policy formulation stage was mixed: there was a clear leadership at the highest level of power (at least for user fee exemption policies and health insurance in Nigeria and Ghana), but the State’s ability to engage the technical and operational levels of government was ineffective; and the State’s coordination efforts when designing the user fee exemption policy was limited.

High-level leadership appeared to have been substantial: ministries took leadership in developing scenarios for the introduction of insurance and user fee exemption schemes [105, 107, 116, 125]. Concurrently, the influence of donors was considered limited. In Ghana, Rwanda, and South Africa, specific laws creating national health insurance schemes were promulgated in the 2000-decade. In Ghana, although there were reports of donors willing to influence the choice of insurance schemes and even “threaten[ing] to march the parliament”, the government had the original bill passed [108]. User fee removal or reduction in Burkina Faso, Senegal and Uganda [107, 113, 117], as well as the PBF policy in Burundi [124] were integrated into countries’ national strategic plans. Therefore, formulation appeared to be government-owned in these countries [130]. Yet, to be able to say that there was ownership at this stage, one also needs to look at the effective engagement of technical and operational levels of government. High-level leadership also needs to be assessed at the policy formulation phase.

Many authors described the technical design of user fee exemption policies as chaotic [107, 112, 113, 126, 131]. The unexpectedness of the decision-making hindered technical inputs from national experts for the development of well-planned policies and their related procedures and implementing rules [107, 112]. The technical level of government did not own the formulation process. As a result, the ability of the State to coordinate actors within public agencies when designing the user fee exemption policy was also limited.

In South Africa and Uganda, ownership of the health insurance policymaking was somehow undermined, but not because of external influence. Other actors involved played a crucial role in negotiating the content of insurance schemes, such as political factions and, more importantly, the private sector [115, 116]. Finally, in Tanzania, politicians played a minor role. Policymaking was mainly the product of top management’s power in the largest mandatory formal sector scheme – the National Health Insurance Fund, a technical agency [116].

### Government ownership at the funding stage

After demonstrating high leadership, governments are expected to secure domestic funding for implementation. Findings were also mixed at the funding stage. While there was evidence of government ownership for health insurance and to a lesser level, user fee exemption, on the contrary, funding for PBF schemes did not appear to come from the governments.

First, authors of most papers highlighted the high dependence of SSA countries covered in this review on external aid (except South Africa, and to a lesser extent, Nigeria) [27]. Some results suggested that this characteristic was conducive of a low ownership of the policymaking process: Tanzania’s and Zambia’s dependence on donors gave influence to the latter in debates over health policies whereas donors’ role was irrelevant in South Africa [119, 131]. However this pattern did not necessarily reflect other aid-dependent countries. In Ghana, external actors played a minimal role, mainly through provision of support to mutual health organization and development of training manuals [105]. The implementation of the nation-scale reforms was the opportunity for the State to mobilize more domestic funding, thereby demonstrating high ownership. In Rwanda, domestic resources were increased as a way to ensure the insurance scheme’s financial sustainability [125].

As far as user fee policies are concerned, the donor dependence thesis was irrelevant as well because most countries introducing user fee exemption largely (Burundi, Ghana, Niger, Uganda) or even exclusively (Burkina Faso) funded the reform implementation through their national budgets [107, 109, 112, 126]. Ridde explained that Burkina Faso demonstrated a particularly high level of commitment by funding through its national budget subsidies covering direct costs [113]. Meessen and colleagues emphasized the use of the Highly Indebted Poor Countries (HIPC) Initiative by governments as “a key instrument to finance the reform” [107]. In a review, despite high levels of external aid in West Africa [107], selected policymakers rated “financial sustainability” as “quite important”, yet less critical than other criteria such as “political leadership”. “Donor money” was the “least important” of all criteria [126]. The case of Ghana was peculiar: the British government provided funding for covering the costs of free deliveries during the first year of implementation before Ghana’s government could take over with domestic funding [109]. However, the situation was often more complex than it looked: policymakers found themselves calling for international donors or NGOs to match the limited domestic funding [107, 112].

In the case of PBF, we found that funding widely came from donors. In Benin and Tanzania, pilot PBF schemes, purchasing of services was entirely covered by donors [128, 132]. In Burundi, the State contributed to half (52%) of the funding for the nation-wide PBF scheme [120]. In Rwanda, we could not find any figure on funding sources in Rusa et al. In a paper about the forthcoming introduction of PBF in Ghana, Burkina Faso, and Tanzania, interviewees, conscious of the policy’s dependence upon donor funding, supported local resources mobilization [110]. They were in favor of finding alternative ways towards creating “a sustainable incentive scheme that could be financed at the local level without external partner support” [110].

### Government ownership at the implementation stage

Overall, the governments’ capacity to effectively engage with and coordinate actors within public agencies to implement these policies was considered limited. User fee exemption policies were implemented with a substantial degree of “improvisation” that illustrated substantial planning deficiencies from the public authorities [112]. In West Africa and Malawi, barriers to the effective implementation were numerous: unpreparedness at the technical, financial and communication levels [112, 131, 133]; incapacity of the health system to sufficiently respond to increasing demand induced by user fee removal; etc. In Burkina Faso, Mali, and Niger these issues did not however impede policy, which was operationalized by national technicians “without any particular form of external assistance” [111]. The case of Niger was symptomatic of insufficient financial planning. The president claimed a “sovereign” decision based on national funding, yet according to authors the State proved unable to meet its financial commitments [112]. Other authors reported that in Mali, a NGO played an instrumental role in operationalizing the user fee exemption for malaria treatment [122]. Malawi for its part reportedly failed to plan and provide adequate funding to cover the cost of the “free” services [131]. In this case, the ability to secure funding at the implementation stage was lacking. Zambia was better equipped for implementing user fee exemption: funds were planned ahead of time to ensure drug provision [134].

Interestingly, the Ugandan government chose to “diffuse ownership of the reforms to the local governments” [117]. However this strategy reportedly “undermined the ability of the national reform group to effectively advocate for and implement the reforms” [117]. Decentralization was also extensively relied upon to implement health insurance in Nigeria and led to important problems. The ability of the central government to transfer leadership to decentralized governments therefore proved insufficient [118, 131]. In Tanzania, communication about the insurance policy was inadequate [130]. These examples might indicate that in Nigeria, Tanzania, and Uganda, the lack of involvement of technical and operational staff was impeding ownership by implementers. On the contrary, in Ghana, the national insurance policy was implemented without substantial issues, and with very limited intervention from external actors [105].

As per the content of the reviewed body of literature, government ownership in PBF policy operationalization appeared to be limited. In Manitu et al.’s paper, some interviewees expressed concerns that implementing PBF would entail the creation of parallel structures, which in turn would lead to ownership issues [135]. In Burundi, until 2014, implementation of PBF schemes lied in the hands of donors and international NGOs [120, 124]. Transfer of “management and stewardship” was supposed to occur between donors and governments, which according to some authors, officially happened [124]. In Benin, the political discourse appeared to be highly supportive of PBF [128] but debates over national scale-up are still ongoing.

Many articles tackled the need to strengthen the State’s ability to coordinate efforts from all stakeholders involved in policy implementation. For example, authors advocated for more collaboration between political leaders and technocrats in undertaking policies aiming at UHC [112, 130]. Sub-Saharan governments’ coordination capacity was seldom assessed in terms of managing external actors. At the time of publishing, coordination mechanisms were not in place in all countries, and when they were, the extent of their coverage was not always optimal [107].

### Government ownership at the evaluation stage

Ownership of evaluating decision-making on policies aiming at UHC appeared to be fairly limited in the majority of papers, but in the case of Burkina Faso we noted the ability of district health teams to come up with innovative ways to report results.

In the case of PBF, donors were portrayed as both the main instigators and the co-implementers (with governments) of the policy. They strongly engaged in the evaluation process as the continuation of their missions. This was true for Burundi and Rwanda in particular [120, 123, 124]: the authors of these papers mentioned the government and the financial and technical partners as undertaking together the evaluation phase and drawing recommendations and conclusions from it, without distinguishing the roles played by each of them. The degree of government ownership was therefore unclear at this stage. In Manitu et al., some interviewees criticized the fact that experts documenting several PBF experiences were the same that promoted the strategy. Authors recommended that the evaluation be carried out by neutral teams [135].

As for user fee exemption, authors described weak evaluation procedures in five countries [107], and a lack of any “basic system to monitor progress”. Government ownership was hampered here not because of some external influence, but because of internal weaknesses. On the contrary, Meda et al. showed that district teams played a leading role in communicating about both the processes and outcomes of the multiple policies that were being implemented [111].

### General results on government ownership

The preliminary identification of ownership indicators and their linkage to policymaking stages proved to be useful strategies to analyze government ownership of policymaking aiming at UHC. The majority of the selected papers (26/35) presented mixed results in terms of government ownership. In other words, in most papers there was evidence of ownership at one or more stages of the policy implementation process but not all (see Additional File 2 for details).

## Discussion

### A critical look into our main results

This scoping review of the peer-reviewed literature demonstrated mixed results about government ownership of health financing policies aiming at UHC. Authors of only five papers provided evidence of ownership at all reviewed policymaking stages.

In the case of insurance and user fee exemption, when emergence and formulation phases were reportedly government-owned, it was due to political leadership expressed at the highest level of governments. These decisions were often highly personalized in order to ensure political election or re-election, and entailed many technical difficulties for most SSA countries. In addition, when results pointed to a lack of governmental ownership, donors were not necessarily responsible for this situation. Also, donors’ intervention was not necessarily undermining ownership: there were multiple reports of government ownership and donors’ influence successfully coexisting.

Our analysis of the PBF policy was more straightforward: as per our review, donors’ involvement at all policymaking stages (as reported in the selected papers) led to limited government ownership. Indeed, there was limited evidence that PBF policymaking processes were government-owned. In the case of PBF as a national policy, as in Burundi and Rwanda, ﻿selected papers did not provide much information about government ownership﻿ – ﻿but other sources may indicate greater ownership for these two countries﻿. In most SSA countries where it is still a pilot policy, apart from political support, the ability of governments to plan for future institutionalization and funding came forward neither in this review, nor in recent publications [136, 137]. Funding of PBF remains largely ensured by donors [138].

Based on these main results, we identify three areas that need to be discussed. First, the observed differences in terms of ownership and donors’ influence between the policies ought to be explained. Second, reports of a lack of external influence on user fee exemption and, to a lesser extent, health insurance, were often hiding contrasting realities. Third, there is a need to further explain the idea that donors’ influence and government ownership may successfully coexist.

#### Attempting to explain differences across the three policies

Why were there differences between health insurance and user fee exemption on one hand, and PBF on the other hand? First, the level of involvement of donors may explain this discrepancy. While there was an explicit push by donors to undertake two health financing reforms in the 2000s (health insurance and user fee exemption), at the emergence stage, they were less proactive at the funding stage: they hardly provided any specific funding for implementation. The supply-side nature of the PBF policy possibly required greater financial mobilization so as to yield faster results in health facilities: PBF pilot schemes were fully funded by donors. On the contrary, health insurance and user fee exemption are demand-side policies and therefore as such, did not necessarily need high disbursements. The low level of external funding for health insurance could be explained by the structure of insurance itself, which relies on both public and private contributions that are later pooled for members’ benefit.

In the case of PBF, each stage of the policymaking process appeared to be substantially influenced by external actors: PBF pilot programs were thoroughly promoted, designed, funded, implemented, and evaluated by donors and NGOs. The high influence by external actors might be explained by the implications of PBF itself, which purportedly entails better monitoring of funded activities – thereby allowing the emergence in SSA countries of systems that would better track aid funding in general [15]. It is likely that donors perceive PBF as fulfilling their goals in a more efficient way than other policies aiming at UHC [137–140].

Second, it may be that, as an analysis of sustainable development policies in Madagascar also highlighted [139], when donors are simultaneously involved (as in the case of PBF) in policy’s emergence, funding, implementation (through technical support provision to the government), and evaluation stages, government ownership of the policymaking process is likely to be undermined [140]. Concurrently, Sjöstedt argued that there are “inherent tensions” between the principles of government ownership and donors voicing their interests and political priorities of their own governments together with “continuously measuring and reporting results” [33].

#### A superficially high degree of government ownership?

Authors described emergence and formulation as government-owned, based on political leadership expressed at the highest level. However, in the case of user fee exemption policies, there were multiple reports of highly personalized decisions made to increase the popularity of their promoters, not to improve people’s access to care. The subsequent technical difficulties demonstrated that the policy process was not was not fully owned. Moreover, the factors influenced decision-making: some authors acknowledged that SSA governments might have favored the adoption of this policy because “it complies with the health policy vision of the country and of the donors” [107]. The balance of power between national and international actors remains to be analyzed more in-depth.

We did find an example where the results on donors’ influence was presented as low while in fact it was fairly high: in Uganda, the P4H consortium was not described as a donor (despite being composed of various multilateral and bilateral agencies) but had a substantial influence as a major advocate and financer of the national health insurance scheme [115]. We also found that the analyses provided by the authors on funding for user fee exemption in SSA countries might have missed the broader picture. Indeed, authors reported that countries provided funding to implement their policies primarily from their own budget [107, 112, 126]. However, when searching for the specific national fund that was used, we found the name of the “Highly Indebted Poor Countries Initiative (HIPC)”, which was reportedly used in four countries [107]. The HIPC used to be an instrument available to the State’s discretion (albeit for social purposes) that is entirely funded by donors [141]. Therefore, even though the decision to use these funds did come from SSA countries’ governments [142], funding could not be labeled as coming from national sources. In fact, this paper showed that the use of the HIPC fund generated a “wait-and-see attitude” on the Ghanaian government’s side in terms of domestic resource mobilization [142]. By taping into this kind of fund, one risks creating more donor dependency instead of more ownership.

#### Successful coexistence of donors’ influence and government ownership

Donors’ intervention was not necessarily undermining ownership. Indeed, in this review, there were multiple reports of donors and governments working hand in hand towards agenda-setting and formulation. The healthcare user fee exemption policy was an interesting case: the policy apparently emerged through a shared vision between external and internal actors in most countries, and it was formulated through strong political will and leadership of the governments [107, 112, 117].

Consistent with this finding, other literature pinpoint that while we cannot speak of “pure imposition” by external actors anymore [143, 144], the transmission to the national level of policies originating from external actors does still happen in the form of a “collaborative interaction” between them and domestic actors [145, 146]. Some authors argue that SSA countries’ elites actively participate in this collaborative policy transfer [147].

### Strengths and limits of the review

This paper represents the first attempt at analyzing the critical features of government ownership in relation to health financing policies aiming at UHC, by looking at each step of the policymaking process in a systematic fashion. This paper addresses a highly relevant topic for scholars and policymakers with interests in the governance of global-national interface, health financing, and universal health coverage. In addition, this review interestingly reports on differentiated findings and unexpected results: these may open new avenues for research

Our review has some limitations. First, as indicated in the introduction, we chose to limit the investigation to the geographic area of Sub-Saharan Africa. While we believe that this restriction has no impact on the richness of data on user fee exemption and health insurance, we are lacking the experience of other results-based financing policies (including conditional cash transfers and output-based aid) outside of our focus continent.

Second, as also noted in the introduction, we were not able to not look at policies aiming at UHC in its multidimensional meaning, given that global attention and efforts have focused on the financial dimension of UHC over the past decade.

Third, results-based financing policies, like PBF, still consist of pilot programs in most SSA countries. We consider that the making of public policies “results partly from a sustainability process, notably through actions implemented as pilot project” [148]. In our review, we described and analyzed PBF as a public policy since governments of these countries have participated in their implementation (by providing the available human and material resources) and demonstrated political will for making them countrywide public policies. Readers should also be aware that PBF is still relatively new in most SSA countries: our results may not represent the long-term picture.

Fourth, we decided not to include grey literature or primary policy documents. Indeed, in spite of their relevance, adding this type of non peer-reviewed literature about government ownership in all SSA countries could not have been done in the same transparent and traceable manner.

### Implications for future research

Future research in the area of government ownership and health financing in developing countries should analyze the historical contexts behind the imbalance of power between the different actors during policy negotiations. Researchers should also investigate the power of national actors themselves, such as exploring how some national actors become themselves policy champions, manage to convince other key actors, and thereby influence formulation.

#### Unpacking the complexity of donors’ influence

Traditionally, external donors have had a major influence on decision-making. De Renzio et al. argue that beyond aid dependency, the history of engagement with donors was instrumental in shaping donors’ authority [47]: the debt and balance of payments crises of the 1970s–1980s prompted many SSA countries to seek financial help from the IFIs. In these countries, “donors soon expanded their influence from macroeconomic policies […], to the process of policymaking itself by the early 2000s” [47]. Such a situation created the conditions of a loss of ownership. However, scholars in health financing policymaking appear to have overlooked these historical accounts. Future research on UHC policymaking should aim at filling this gap [60].

Furthermore, donors did not always show a united influence. There was evidence that donors negotiated among themselves to influence the course of policy emergence and formulation. Tanzania’s case was emblematic: in two instances, two groups of donors confronted each other on the preferred financial arrangement (health insurance vs. tax funding) and on the introduction of PBF [116, 140]. Amidst these negotiations, the Tanzanian government was unable to take on the leading role it was supposed to play. In Benin, tensions between the Belgian and World Bank PBF schemes also appeared to have emerged [128]. In light of these findings, future investigations need to explore how donors coordinate policies aiming at UHC.

#### Need for further investigation of the roles played by national actors

Beyond looking at donors’ influence on emergence, it is important to analyze the path of national actors identifying with a given policy and taking the lead in pushing it forward to secure policy formulation [105, 113, 118, 126, 149]. However, we could find little information about the ways these national actors were organized and the extent to which they identified with a policy that emerged externally. A recent paper identified the lack of “national policy entrepreneurs” as the main reason for the “failed” emergence of PBF in Chad [150]. Analyzing the paths of national “policy champions” is an avenue for future research.

Besides political elites, other factors may influence policymaking processes. For instance, a context of chronic political instability can undermine the implementation and financial sustainability of policies. None of the papers addressed this particular influence. The role played by other non-state actors and scientific evidence was scarcely tackled in the selected papers. We showed that other non-state actors such as NGOs and countries’ private sector have an impact on governments’ decisions, as this was the case in Mali (on user fee exemption), South Africa, and Uganda (on health insurance); yet the action and interests of such non-state actors (whether they are domestic or foreign), increasingly financially supported by bilateral donors [151, 152], may conflict with those of the governments and undermine the position of the State [153, 154]. One paper also highlighted the influence on policymaking of scientific and experts’ evidence produced and disseminated by donors [127]: this finding concurs with other works at both national and global scales [155–157]. Future research should address the influence of these non-state actors as well as the category of “non-human” drivers of policymaking.

### Implications for practice: the way towards effective government ownership of policies aiming at UHC

Based on this review, it is possible to formulate a few recommendations. First, we advise that pilot schemes be carefully planned by clarifying the roles played by each category of actors, notably by distinguishing political advocacy, funding, technical support for policy implementation, and evaluation. We suggest using donor funding for i) supporting the development of national UHC policies and infrastructure, ii) building and strengthening long-term State capacities in coordinating the different actors involved at any stage of the policymaking, and iii) providing ideas for domestic funding mobilization (e.g., increased taxation of international companies established in the country).

## Additional file

Additional file 1: Search strategy. (DOCX 13 kb)
Additional file 2: Main findings from the review about government ownership. Legend: *Red color* indicates lack of evidence of government ownership (lack evidence of ownership indicators based on selected papers’ findings); *yellow* color indicates mixed evidence of government ownership (mixed evidence); *green color* indicates evidence of government ownership (evidence). (DOCX 160 kb)

## Abbreviations

CCTs: Conditional cash transfers
IFIs: International Financial Institutions
NGOs: Non-governmental organizations
PBF: Performance-based financing
RBF: Results-based financing
UHC: Universal health coverage
WHO: World Health Organization
