# Supplementary material for: Health financing policies in Sub-Saharan Africa: government ownership or donors’ influence? A scoping review of policymaking processes
Source: Glob Health Res Policy. 2017 Aug 8;2:23. doi: 10.1186/s41256-017-0043-x (PMC5683243; doi:10.1186/s41256-017-0043-x)
Supplement: Additional file 1: — Search strategy. (DOCX 13 kb) [file 41256_2017_43_MOESM1_ESM.docx]

**Additional file 1: Search strategy**

The following keywords were used:

(Angola OR Benin OR Botswana OR Burkina Faso OR Burundi OR Cap Verde OR Cabo Verde OR Cameroon OR Central African Republic OR Chad OR Comoros OR Congo, Dem. Rep. OR Democratic Republic of Congo OR Democratic Republic of the Congo OR Republic of Congo OR Congo, Rep. OR Cote d'Ivoire OR Eritrea OR Ethiopia OR Gabon OR The Gambia OR Ghana OR Guinea OR Guinea-Bissau OR Kenya OR Lesotho OR Liberia OR Malawi OR Mali OR Mauritania OR Mauritius OR Maurice OR Mozambique OR Namibia OR Niger OR Nigeria OR Rwanda OR Sao Tome and Principe OR Senegal OR Seychelles OR Sierra Leone OR Somalia OR South Africa OR South Sudan OR Sudan OR Soudan OR Swaziland OR Tanzania OR Togo OR Uganda OR Zambia OR Zimbabwe OR Africa OR Subsaharan Africa OR sub-Sahara)

AND (universal health coverage OR health insurance OR health micro-insurance OR performance-based financing OR results-based financing OR user-fee exemption OR user-fee removal OR conditional cash transfer OR conditional cash payment OR community-based insurance OR mutuelle)

AND (country ownership OR country-owned OR national ownership OR government ownership OR state ownership OR country buy-in OR government buy-in OR country acceptance OR state acceptance OR national policy OR national strategy OR national plan OR national strategic plan OR “adapt* to country”)
