# Supplementary material for: Health financing policies in Sub-Saharan Africa: government ownership or donors’ influence? A scoping review of policymaking processes
Source: Glob Health Res Policy. 2017 Aug 8;2:23. doi: 10.1186/s41256-017-0043-x (PMC5683243; doi:10.1186/s41256-017-0043-x)
Supplement: Additional file 2: — Main findings from the review about government ownership. Legend: Red color indicates lack of evidence of government ownership (lack evidence of ownership indicators based on selected papers’ findings); yellow color indicates mixed evidence of government ownership (mixed evidence); green color indicates evidence of government ownership (evidence). (DOCX 160 kb) [file 41256_2017_43_MOESM2_ESM.docx]

| **Article details** | **Emergence** | **Formulation** | **Funding** | **Implementation** | **Evaluation** | **Result** |
| --- | --- | --- | --- | --- | --- | --- |
| **Agyepong et al 2008** | NA | Bill passed by the government | Proven capacity to mobilize national resources for contributing to finance the policy | Proven capacity to act and coordinate actors within public agencies; some technical support from external actors | NA | Yes |
| **Atim 2011** | NA | "Tension between political leadership and the technical design group or ‘technocrats'" | Insufficient resource mobilization to cope with increased use | NA | NA | No |
| **Basaza et al 2013** | Political will demonstrated at the highest level of government | Influence of the private sector in the choice of the insurance scheme | Compared to the government, donors provide more than twice as much funding for public health expenditures | Lack of capacity to coordinate actors within public agencies | NA | Mixed |
| **Chimhutu et al 2014** | Donor's influence preceded over political will of the gov't | NA | Locally-funded scheme | NA | NA | Mixed |
| **Chirwa et al 2013** | Government's and Western donors' conjoint will; strong consensus between the government and the main faith-based provider | Insufficient engagement at technical and operational levels of government | External funding; no evidence of increased resource mobilization | Ineffective engagement at technical and operational levels of government; lack of capacity to coordinate actors within public agencies | Inadequate monitoring and evaluation system | Mixed |
| **Falisse et al 2012** | Donor's influence preceded over political will of the gov't | Ministerial will to undertake national scale-up of the PBF schemes | The state of Burundi contributes to 52% of PBF funding | Government took over the management and stewardship of existing projects | Monitoring and evaluation undertaken by private actors | Mixed |
| **Falisse et al 2014** | *idem* | National policy following "a series of discussions between the donors […] and the government" | NA | NA | NA | Mixed |
| **Fox et al 2014** | No data on the government's role | World Bank's approach to PBF | World Bank funding | Implementation led by international NGOs | NA | No |
| **Gilson et al 2003** | Zambia: Donor's influence preceded over political will of the gov't South Africa: Evidence of national leadership | Strong leadership by both Ministers of Health | Zambia: External funding; no evidence of increased resource mobilization South Africa: National treasury mobilized | Engagement at technical and operational levels of government; proven capacity to coordinate actors within public agencies | NA | Yes (South Africa) Mixed (Zambia) |
| **Gilson et al 2012** | Tanzania: Donors' influence  South Africa: Evidence of national leadership | Tanzania: Lack of capacity to coordinate actors within public agencies | Tanzania: No data except about aid dependency South Africa: domestic funding | NA | NA | Yes (South Africa) Mixed (Tanzania) |
| **Kajula et al 2004** | Donors' influence and political will during electoral campaigns | Highly personalized decision; at the same time, central government attempting to “diffuse ownership of the reforms to local governments" | No evidence of increased resource mobilization | Ineffective engagement at technical and operational levels of government | NA | Mixed |
| **Kirigia & Diarra-Nama 2008** | NA | NA | Aid dependency | NA | NA | No |
| **Kusi-Ampofo et al 2015** | Donors' influence and political will | NA | NA | NA | NA | Mixed |
| **Manitu et al 2015** | *idem* | NA | Fragmented pools; lack of capacity to coordinate donors' projects | Creation of parallel structures undermining public health system | "Several PBF experiences were documented by experts who promote the strategy" | Mixed |
| **Masiye et al 2010** | Political will at the highest-level | Decision consistent with previous national policies | Funding provided by a donor for the "transitional period", but then the Zambian Government itself made additional resources available to support the policy | Effective engagement of technical and operational levels of government; strategic planning | NA | Mixed |
| **Mbaye et al 2013** | Political will at the highest-level during election campaigns | Ineffective engagement at technical and operational levels of government | No external funding, but no evidence of increased resource mobilization | NA | NA | Mixed |
| **McIntyre et al 2013** | Political will at the highest-level | Ineffective engagement at technical and operational levels of government | Fragmented pools, unproven ability to mobilize funding | Nigeria: complications due to decentralization Malawi and Tanzania: ineffective engagement of technical and operational levels of government | NA | No |
| **Meda et al 2011** | NA | NA | Use of local resources for contributing to finance the policy | Dynamic management and strategic planning | Lessons shared in a quarterly bulletin | Yes |
| **Meessen 2011** | Donors' influence and political will at the highest-level during election campaigns | Expedited policymaking; ineffective engagement at technical and operational levels of government | National resource mobilization for contributing to finance the policy | Ineffective engagement of technical and operational levels of government; lack of capacity to coordinate actors within public agencies | Weak monitoring and evaluation system in five countries | Mixed |
| **Nabyonga-Orem et al 2014** | Donors' influence (divided) and political will at the highest-level during election campaigns | NA | Lack of capacity to coordinate actors within public agencies | NA | NA | Mixed |
| **Nyandekwe et al 2014** | Political will and leadership demonstrated at the highest level of government | Bill passed by the government | Increased mobilization of domestic resources to ensure financial independency for the insurance scheme | Effective engagement of technical and operational levels of government; strategic planning | NA | Yes |
| **Olivier de Sardan et al 2012** | Donors' influence and political will at the highest-level during election campaigns | Expedited policymaking; ineffective engagement at technical and operational levels of government | Use of national resources for contributing to finance the policy | Mali & Niger: Lack of capacity to act and coordinate actors within public agencies | NA | Mixed |
| **Onoka et al 2014** | Political will and leadership demonstrated at the highest level of government | Effective engagement of technical and operational levels of government; ability to "galvinize interest" from all actors | NA | Successful engagement of health maintenance organizations but lack of support from states | NA | Yes |
| **Paul et al 2014** | Donor's influence preceded over political will of the gov't | "Field actors" not consulted during formulation | External funding; no evidence of increased resource mobilization | Policy is welcomed by implementers but "they do not have a sense of ownership about it" | NA | Mixed |
| **Peerenboom et al 2014** | NA | Donor's influence preceded over political will of the gov't | External funding; no evidence of increased resource mobilization | Effective engagement of technical and operational levels of government; simplification of administrative procedures | Donors and the government making recommendations for better coordination in concert | Mixed |
| **Ponsar et al 2011** | NA | Donor's influence appear to have preceded over political will of the government | External funding; no evidence of increased resource mobilization | Joint supervision of activities by the Ministry of health and an international NGO | NA | Mixed |
| **Ridde 2011** | Donors' influence and political will at the highest-level | Effective engagement of technical and operational levels of government: setting up a technical committee to define the policy | Funding of the policy through the national budget | NA | NA | Mixed |
| **Ridde et al 2012** | Donors' influence and political will at the highest-level | National civil society influencing decision-making in Senegal | In the majority of countries: evidence state financing; Mali, Niger, Togo: state and partners funding; "ineffective and poorly funded coordination systems" | "Almost no implementation guides, lack of support measures"; communication plans were rarely carried out, lack of engagement of health workers | "Almost no evaluation system" | Mixed |
| **Rusa et al. 2009** | Donor's influence preceded over political will of the government | Decision taken by the Minister of health in concert with the Belgian Technical Cooperation | NA | Apparent effective engagement of technical and operational levels of government | Donors and the government making recommendations for better coordination in concert | Mixed |
| **Seddoh et Akor 2012** | "Technical experts, civil society, academicians and politicians all had significant influence on setting the health insurance agenda" | Political will and leadership demonstrated at the highest level of government | NA | National decision to use externally-provisioned fund | NA | Mixed |
| **Thomas & Gilson 2004** | Political will and leadership demonstrated at the highest level of government | "Failure to develop a policy around national or social health insurance with the successful adoption of regulation of private health insurance" | NA | NA | NA | Yes |
| **Torbica et al 2014** | Political will at the highest-level during election campaigns | Expedited policymaking; ineffective engagement at technical and operational levels of government | Financial sustainability and donors' funding not considered very important by policymakers | Lack of consideration for strategic planning and institutionalization | NA | Mixed |
| **Witter et al 2013a** | Political will and leadership demonstrated at the highest level of government | Decision consistent with previous national policies | Lack of financial visibility: "As the donor-funded initiatives funds are spent independently, the [Ministry] does not have information on disbursements" | NA | NA | Mixed |
| **Witter et al 2013b** | Donors' influence and political will at the highest-level | Decision taken by the Minister of health in concert with the government of the United Kingdom | External funding for covering the costs during first year of implementation before mobilizing domestic funds | "Limited stakeholder consultation. No costing was done prior to introduction" | NA | Mixed |
| **Ye et al 2014** | Donor's influence preceded over political will of the government | NA | Health providers worried about financial sustainability; willing to create a sustainable incentive scheme that could be financed at the local level | NA | NA | Mixed |
